# Analysis of Ingredients of Supplements in the National Institutes of Health Supplement Database Marketed as Containing a Novel Alternative to Anabolic Steroids

**DOI:** 10.1001/jamanetworkopen.2020.2818

**Published:** 2020-04-15

**Authors:** Pieter A. Cohen, Joshua Sharfstein, Angélique Kamugisha, Céline Vanhee

**Affiliations:** 1 Department of Medicine, Cambridge Health Alliance, Somerville, Massachusetts; 2 Harvard Medical School, Boston, Massachusetts; 3 Department of Health Policy and Management, Johns Hopkins Bloomberg School of Public Health, Baltimore, Maryland; 4 Department of Medicines and Health Products, Sciensano, Brussels, Belgium

## Abstract

This case series analyzes whether supplements categorized as containing novel ingredients in the National Institutes of Health Dietary Supplement Label Database are accurately labeled.

## Introduction

Dietary supplements are estimated to be responsible for tens of thousands of emergency department visits each year in the US.^1^ To identify potentially dangerous supplements and manage their risks, clinicians and consumers require access to accurate information about supplement ingredients. In an effort to provide a resource for researchers, clinicians, and the public, the National Institutes of Health (NIH) maintains an online searchable database of supplement labels.^2^ To our knowledge, whether supplements categorized as containing novel ingredients in the NIH database are accurately labeled has not been previously studied.

In October 2019, the US Food and Drug Administration (FDA) placed 5-alpha-hydroxy-laxogenin, a synthetic analog of a plant steroid that is marketed as a natural alternative to anabolic steroids,^3^ on the Dietary Supplement Ingredient Advisory List to alert the public that it does not appear to be a lawful supplement ingredient.^4^ We analyzed the actual contents of supplements categorized as containing 5-alpha-hydroxy-laxogenin in the NIH database to determine whether they are accurately labeled.

## Methods

In this case series, supplements categorized as containing 5-alpha-hydroxy-laxogenin in the NIH Dietary Supplement Label Database^2^ were purchased online in November 2018 and analyzed from January to June 2019 for their chemical contents (excluding expedients, minerals, and plant species) according to previously described methods (eAppendix in the Supplement).^5^ Quantities were considered accurately labeled if they were within 20% of the labeled quantity. Data analysis was conducted with Excel 2016 (Microsoft Corp). The statistical prerequisite established was the determination of goodness of fit (ie, *R*^2^ ≥ 0.95) to conclude that linear calibration lines were fit for estimation purposes within the chosen concentration interval.

## Results

A total of 6 supplements were categorized in the NIH database as containing 5-alpha-hydroxy-laxogenin; 4 (67%) were available for purchase (1 [17%] was out of stock; 1 [17%] was discontinued). In total, 16 ingredients, excluding expedients, minerals, and plant species, were declared on the 4 product labels.

Compared with their actual chemical contents, no product was accurately labeled (Table). Of the 16 ingredients, 6 (38%) were not detected in the supplements. Only 4 of 14 ingredients (29%) with quantities provided on the label were present in the specified amounts. Quantities detected ranged from less than 5% to 109% of what was listed on the label. Two products contained an ingredient not declared on the label.

**Table.** Label Information and Actual Contents of Dietary Supplements Categorized as Containing 5-Alpha-Hydroxy-Laxogenin

Product code	Declared ingredient and quantity, mg	Detected ingredients, mean (SD), mg			Declared ingredient not detected^b^	Detected ingredient not declared on the label and quantity, mean (SD), mg
		Quantity on label^a^		Without quantity provided on label		
		Accurate	Inaccurate			
A	5-alpha-hydroxy-laxogenin, 90	NA	NA	NA	5-alpha-hydroxy-laxogenin	NA
	GABA, NP	NA	NA	GABA, 177.7 (0.2)	NA	NA
	Folic acid, 0.2	NA	NA	NA	Folic acid	NA
	Melatonin, 3.0	Melatonin, 3.3 (3.1)	NA	NA	NA	NA
	Phenibut, NP	NA	NA	Phenibut, 72.1 (0.1)	NA	NA
	Vitamin B_6_, 6	NA	Pyridoxine, vitamin B_6_, <0.5^c^	NA	NA	NA
	Vitamin B_12_, 0.010	NA	NA	NA	Vitamin B_12_	NA
	Vitamin D, 0.013	NA	NA	NA	Vitamin D	NA
B	5-alpha-hydroxy-laxogenin, 50	NA	NA	NA	5-alpha-hydroxy-laxogenin	NA
	NA	NA	NA	NA	NA	Diosgenin, 118 (2.8)^d^
C	N-acetylcysteine, 250	N-acetyl cysteine, 235.7 (4.7)	NA	NA	NA	NA
	Androst-3,5-diene-7,17-dione, 75	Androst-3,5-diene-7,17-dione, 71.8 (4.3)	NA	NA	NA	NA
	5-alpha-hydroxy-Laxogenin, 50	NA	NA	NA	5-alpha-hydroxy-laxogenin	NA
	NA	NA	NA	NA	NA	Diosgenin, 81.1 (5.6)^d^
D	5alpha-hydroxy-laxogenin, 105	NA	5-alpha-hydroxy-laxogenin, 50.5 (1.0)	NA	NA	NA
	β-ecdysterone, 105	NA	β-ecdysterone, 58.7 (8.8)	NA	NA	NA
	Bioperine, 30^e^	NA	Piperine, 0.4 (8.3)	NA	NA	NA
	7-keto DHEA, 60	7-keto DHEA, 58.1 (3.4)	NA	NA	NA	NA

Abbreviations: DHEA, dehydroepiandrosterone; GABA, γ-aminobutyric acid; NA, not applicable; NP, none provided.

^a^ Accurate indicates that the amount of the ingredient detected was within 20% of the amount listed on the label. If the difference between the amount on the label and the amount detected exceeded 20%, the ingredient was classified inaccurate. The quantities of labeled and nonlabeled ingredients are expressed in mg per maximum daily serving size recommended on the label. Maximum daily serving size was calculated by multiplying the quantity of an ingredient found in an individual serving size by the maximum servings per day recommended on the label.

^b^ The limit of detection of 5-alpha-hydroxy-laxogenin was less than 0.02 mg of this molecule per capsule of 500 mg powder (ie, less than what is present in 1 capsule of all samples). The limit of detection of folic acid, vitamin B_12_, and vitamin D (ie, ergocalciferol and cholecalciferol) was lower than 0.005 mg of this molecule per capsule.

^c^ Owing to the small detectable amount of pyridoxine in the sample, SD could not be calculated.

^d^ The label of the product listed a plant that may contain small amounts of diosgenin as a constituent (ie, sample B was labeled as containing an extract of *Smilax sieboldii* while sample C was labeled as containing extracts from both saw palmetto and *Tribulus terrestris*).

^e^ Bioperine is a patented extract obtained from black pepper fruits containing at least 95% piperine.

Only 1 product (25%) contained 5-alpha-hydroxy-laxogenin, an ingredient with purported anabolic effects in rats that, to our knowledge, has not been studied in humans. The supplements contained phenibut, an unapproved drug with abuse potential that is used in Russia to treat a variety of neurologic conditions; arimistane, a designer steroid that may function as an aromatase inhibitor; 7-keto dehydroepiandrosterone, a minor metabolite of dehydroepiandrosterone; ecdysterone, a steroid-like compound found in many insects and plants that may have anabolic effects in humans; and diosgenin, a steroid-like compound found in trace amounts in several plants and synthetically produced for use in the industrial production of some commercial steroids (Figure). None of these ingredients has an FDA-approved indication.

**Figure.**
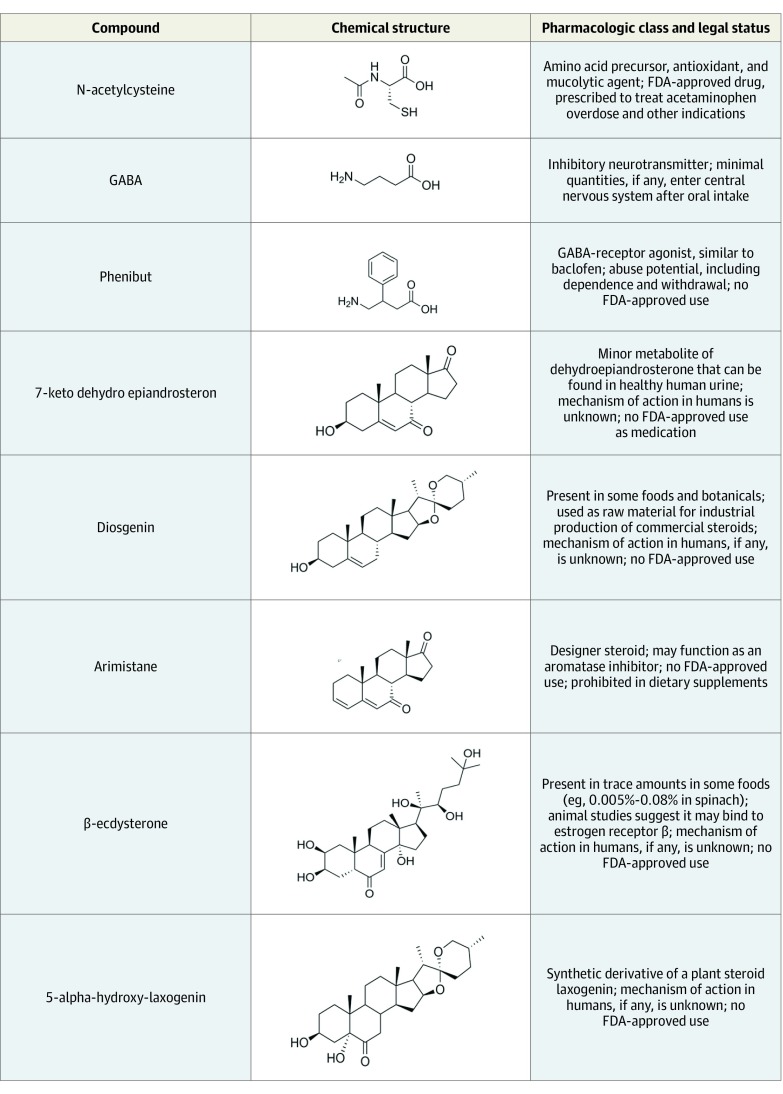
Pharmacologic Class, Chemical Structure, and Legal Status of Compounds Detected in Dietary Supplements Categorized as Containing 5-Alpha-Hydroxy-Laxogenin Abbreviations: FDA, US Food and Drug Administration; GABA, γ-aminobutyric acid.

## Discussion

Supplements categorized in the NIH supplement database as containing 5-alpha-hydroxy-laxogenin were inaccurately labeled and contained a variety of potentially pharmaceutically active compounds with unpredictable health effects. The NIH database contains the disclaimer that “label information has not been verified or checked for conformity with existing US Food and Drug Administration (FDA) regulations.”^2^ However, this statement does not accurately capture the breadth of gaps in the database, including supplements that contain ingredients not listed on the label, do not contain ingredients declared on the label, provide inaccurate quantities of ingredients on the label, and contain prohibited ingredients.

Our study was limited because we analyzed only supplements categorized as containing 1 new ingredient at 1 point. Ingredients may vary over time within the same product.^6^

Our analysis suggests that consumers and clinicians cannot obtain accurate information on novel supplement ingredients with the NIH supplement database. Future efforts to design and update supplement databases should take steps to assure accurate results.

## Notes

Supplement. **eAppendix.** Supplemental Methods **eReferences.**
