# Supplementary material for: Analysis of Ingredients of Supplements in the National Institutes of Health Supplement Database Marketed as Containing a Novel Alternative to Anabolic Steroids
Source: JAMA Netw Open. 2020 Apr 15;3(4):e202818. doi: 10.1001/jamanetworkopen.2020.2818 (PMC7160690; doi:10.1001/jamanetworkopen.2020.2818)
Supplement: Supplement. — eAppendix. Supplemental Methods eReferences. [file jamanetwopen-3-e202818-s001.pdf]

Supplementary Online Content

Cohen PA, Sharfstein J, Kamugisha A, Vanhee C. Analysis of ingredients of supplements in the National Institutes of Health supplement database marketed as containing a novel alternative to anabolic steriods. *JAMA Netw Open*. 2020;3(4):e202818. doi:10.1001/jamanetworkopen.2020.2818

eAppendix. Supplemental Methods
eReferences.

This supplementary material has been provided by the authors to give readers additional information about their work.

eAppendix. Supplemental Methods

Chemicals and Reagents

Acetonitrile and methanol (mass spectrometry (MS) grade) and analytical grade formic acid were purchased from Biosolve (Valkenswaard, the Netherlands). Water was obtained using a milliQ-Gradient A10 system (Millipore, Billerica, USA).

Reference standards for melatonin, pyridoxine, diosgenin, gamma-aminobutyric acid (GABA), N-acetyl cysteine, β-ecdysone and piperine were bought from Sigma Aldrich (Saint Louis, MO, USA). Androst-3,5-diene-7,17-dione and 7-keto dehydroepiandrosterone (7-keto DHEA) were purchased from Cayman Chemical (Ann Arbor, Michigan, USA). Phenibut originated form TRC Canada (North York, ON, Canada) while 5alpha-hydroxylaxogenin was synthesised by abcr GmbH (Karlsruhe, Germany). All reference standards were solubilised either in methanol or in water (GABA, vitamin B6 under the form of pyridoxine, phenibut and N-acetyl cysteine) and served to concur the obtained screening data or were utilised for quantification purposes.

Sample Preparation for Mass Spectrometry

The food supplements were subjected to analysis by non-targeted gas chromatography mass spectrometry (GC-MS) and non-targeted liquid chromatography-tandem mass spectrometry (LC-MS/MS). For each supplement, a mixture of the content of 10 capsules was made and 30 mg of this mixture was solubilised in either 10 mL of methanol or 10 mL of water (only for the LC-MS/MS method for hydrophilic compounds) and sonicated for 15 minutes. Next, the methanolic extraction was filtered through a 0.2 µm Polytetrafluoroethylene (PTFE) filter prior to analysis by GC-MS or the general LC-MS/MS methodology. Additionally, also the water extraction was filtered through the same type of filter prior to analysis by the alternative LC-MS/MS methodology, utilised to screen for hydrophilic molecules with a small Mw, e.g. GABA.

Gas Chromatography Mass Spectrometry (GC-MS)

The analyses were performed on an Agilent 7890A gas chromatograph coupled to an Agilent 5975 mass selective detector. The Column VF-5ms + 10 m EZ-guard was purchased from Agilent technologies (Santa Clara, CA, USA).

Full automation was achieved using Agilent MSD ChemStation data acquisition and data handling software. Injections were made in the split mode (3.3:1) at an injection port temperature of 280°C. The column oven temperature was initially set to 80 °C for 2 min and then raised at 15 °C/min to 280 °C and held for 17 min, followed by a raise of 10 °C/ min to 310 °C and held for 20 minutes. The total run time was 55 min. The temperatures of the interface, the source and the quad interface were 280, 230 and 150 °C, respectively. The compounds were separated on an Agilent column (30 m x 0,25 mm x 0,25 µm film thickness). High-purity helium was used as the carrier gas with flow rate of 1 mL/min and the column head pressure was set at 93.169 kPa. The MS was operated in electron ionisation with electron energy of 70 eV, a source temperature of 230 °C and a ion multiplier gain of 1.765 kV. Data were acquired in full scan mode of m/z 25 to 600.

Liquid Chromatography-Tandem Mass Spectrometry (LC-MS/MS)

The solubilised suspected sample was first subjected to analysis on a Dionex UltiMate 3000 Rapid Separation LC (RSLC) system (Thermo Scientific, Sunnyvale, CA, USA) coupled to an amaZon™ speed ETD mass spectrometer (Bruker Daltonics, Bremen, Germany). The instrument system was calibrated using the manufacturer's calibration mixture, and the mass accuracy was determined to be < 0.1 Da during the period of analysis. A sample volume of 1 µL was injected onto the system. The chromatographic separation was performed as previously described (1). This general ultra-high performance LC (UPLC) method was shown to be suitable for rapid screening of unknown samples. Briefly, the mobile phase consisted of 0.1% formic acid in water (A) and 0.1% formic acid in acetonitrile (B). A linear gradient of 1% B to 99% B was accomplished in 9 min, followed by an isocratic elution for 2 min and 2 min re-equilibration at 1% B. The flow rate was 0.5 mL/min and the column (an Acquity UPLC BEH C18 Column (150 × 2.1 mm, 1.7 µm particle size) (Waters, Milford, MA, USA)) was set at 45°C.

The mass spectrometer settings were similar to what has been previously described (1). Small alterations were made to the MS mass range (100–1000 m/z). The LC and MS data were analysed by Compass Data Analysis 4.2 (Bruker Daltonics, Bremen, Germany) and the LC-MS/MS spectra were compared to different libraries, including the home made library, incorporating a total of about 5000 MS/MS spectra. A compound was considered present if the difference in retention was less than or equal to 0.5 min (compared with the retention time of the reference standard of this compound), m/z of the precursor ion is equal to this in the in-house library

© 2020 Cohen PE et al. *JAMA Network Open.*

(error tolerance: 0.3 Da) and the MS2 spectrum matches at least 85% with the reference spectrum (fragment ions and their relative intensities) of the in-house library (2).

Liquid Chromatography-Tandem Mass Spectrometry (LC-MS/MS) for hydrophilic small Mw compounds (80-300 m/z)

High resolution accurate mass (HRAM) LC-MS/MS analyses were carried out on Thermo Scientific™ Vanquish™ ultra-high performance liquid chromatography (UHPLC) system coupled to a Q Exactive™ focus orbitrap mass spectrometer (Thermo Fisher Scientific, Bremen, Germany) according to Vanhee et al. (3).

The unknown samples and the reference standards were separated on an Acquity UPLC HSS T3 column (100 mm × 2.1 µm, 1.8 µm particle size) (Waters, Milford, MA, USA). The LC methodology was as follows: isocratic elution for 1 minute of 100% mobile phase A (0.1% of formic acid in water) at a constant flow rate of 0.4 mL/min and a column temperature of 40 °C. The gradient started from 0 to 24% B (0.1% formic acid in acetonitrile) in 9 min, increased to 60% B in the next min, then was kept at 60% B for 3 min, and finally was increased to 98% B for one minute, followed by a 2 minutes re-equilibration step (total run time: 17 minutes).

The Q-Exactive focus mass spectrometer was operated in alternating positive and negative ion mode with subsequent alternating full MS scans of the precursor ions and all ion fragmentation scan (AIF) in which the precursor ions were fragmented by higher energy collisional dissociation (HCD). Both scan types were performed with 70,000 resolution (at m/z 200) with the maximum ion injection time set at 50 milliseconds. The m/z range for the full MS scans was 80–300, and the m/z range for AIF scans was 80–300. The target value for the full MS scans was 106 ions and the target value for the AIF scans was 3 × 106 ions. The normalised HCD collision energy was set at 30%. The heated electrospray ionization (HESI) conditions were as follows: spray voltage: 3.5 kV (positive mode) and 2.5 KV (negative mode); sheath gas flow rate: 51 arb; auxiliary gas flow rate: 13 arb; heated capillary temperature: 264 °C; S-lens RF level: 50 V. Nitrogen was used for spray stabilization and as the collision gas in the C-trap.

All data were collected in profile mode and were acquired and processed by using the Thermo Xcalibur 4.0 software (Thermo Fisher Scientific, Bremen, Germany). A compound was considered present if the difference in retention was less than or equal to 0.5 min (compared with the retention time of the reference standard of this compound), m/z of the precursor ion is equal to the one obtained with the reference standard (error tolerance: 10 ppm) and the MS2 spectrum displayed a very high level of similarity to the MS spectrum of the reference standard (fragment ions and their relative intensities). This methodology, put in place to tackle the low sensitivity of our workhorse LC-MS/MS methodology, has been used for hydrophilic molecules with an MW bellow 200 Da, such as GABA, pyridoxine, N-acetyl cysteine and phenibut.

Quantity Estimation

The quantity of molecule present in the food supplement was estimated based on the extracted ion chromatograms, obtained from the analyte present in the sample compared to relative response of the reference standard.

The linearity of the response of the reference standard was assessed by analysis the R2 values obtained for a 5 point calibration. With all R2 values above 0.96, we concluded that linear calibration lines were fit for estimation purpose, within the chosen concentration interval. The sample was subjected to serial dilution until a similar response area within the linear response interval was obtained.

© 2020 Cohen PE et al. *JAMA Network Open.*

eReferences

1. Vanhee C, Tuenter E, Kamugisha A et al. Identification and quantification methodology for the analysis of suspected illegal dietary supplements: reference standard or no reference standard, that's the question [published online April 20, 2018]. *J Forensic Tox Pharm*. 2017; 6:2. doi: 10.4172/2325-9841.1000156

2. Tie Y, Vanhee C, Deconinck E, Adams E. Development and validation of chromatographic methods for screening and subsequent quantification of suspected illegal antimicrobial drugs encountered on the Belgian market. *Talanta*. 2019;194:876-884. doi:10.1016/j.talanta.2018.10.078.

3. Vanhee C, Francotte A, Janvier S, Deconinck E. The occurrence of putative cognitive enhancing research peptides in seized pharmaceutical preparations: an incentive for controlling agencies to prepare for future encounters of the kind [accepted October 11, 2019]. *Drug Test Anal*. doi:10.1002/dta.2717.

© 2020 Cohen PE et al. *JAMA Network Open.*
